# Resveratrol affects *in vitro* rumen fermentation, methane production and prokaryotic community composition in a time‐ and diet‐specific manner

**DOI:** 10.1111/1751-7915.13566

**Published:** 2020-04-15

**Authors:** Tao Ma, W. Wu, Y. Tu, N. Zhang, Q. Diao

**Affiliations:** ^1^ Key laboratory of Feed Biotechnology of the Ministry of Agriculture and Rural Affairs/Beijing Key Laboratory for Dairy Cow Nutrition Feed Research Institute Chinese Academy of Agricultural Sciences Beijing China; ^2^ College of Animal Science and Technology Gansu Agricultural University Lanzhou, Gansu China

## Abstract

This study aimed to investigate the effect of resveratrol on methane production, rumen fermentation and microbial composition under high‐concentrate (HC) and high‐forage (HF) diets using the *in vitro* fermentation system. A total of 25 mg of resveratrol was supplemented into 300 mg of either HC or HF diet. Methane production, total volatile fatty acid (VFA) concentration, molar proportion of VFA, metabolites of resveratrol and prokaryotic community composition were measured after 12 and 24 h of *in vitro* fermentation. Resveratrol reduced methane production (ml per mg of dry matter degraded) by 41% and 60% under both HC and HF diets (*P* < 0.001), respectively, and this result could be associated with the lower abundance of *Methanobrevibacter* (*P* < 0.001) in response to resveratrol. The molar proportion of propionate was significantly higher in the resveratrol group only under the HC diet (*P* = 0.045). The relative abundance of 10 bacterial genera was affected by the three‐way interaction of treatment, diet and time (*P* < 0.05). Resveratrol was partly converted to dihydroresveratrol after 24 h of fermentation, and its degradation could be associated with microbes belonging to the order *Coriobacteriales*. Our results suggest that multiple factors (e.g. diet and time) should be considered in animal experiments to test the effect of polyphenol or other plant extracts on rumen fermentation, methane emission and microbial composition.

## Graphical abstract

Resveratrol inhibited in‐vitro methane production, which could be associated with the lower abundance of Methanobrevibacter. Resveratrol was partly converted to dihydroresveratrol after 24 h of fermentation.

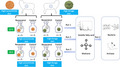

## Introduction

Although methanogenesis is a fundamental rumen metabolic process, it could be responsible for the 2–12% of gross energy loss in feed (Johnson and Johnson, 1995) and contributes to 11–17% of the global greenhouse gas emissions (Beauchemin *et al.*, 2009; Goel and Makkar, 2012). In this regard, great efforts have been made to manipulate rumen fermentation to mitigate methane production (Patra *et al.*, 2017; Henderson *et al.*, 2018). Natural components originating from plants are promising anti‐methanogenic compounds, including essential oils, saponins, tannins and other polyphenols (Bodas *et al.*, 2012; Jayanegara*et al.*, 2014; Cobellis *et al.*, 2016). As a polyphenol phytoalexin, resveratrol (3,5,4’‐trihydroxy‐trans‐stilbene) is found in a variety of plants such as grapes and peanuts (Walle *et al.*, 2004). Resveratrol has received considerable attention because of its wide spectrum of biological functions including antioxidant, anti‐inflammatory and antimicrobial activities (Baur and Sinclair, 2006; Jung *et al.*, 2009). We speculate that resveratrol may hinder methane production by inhibiting the major methanogens (e.g. *Methanobrevibacter*) in the rumen.

To our knowledge, studies are lacking on the effect of polyphenols on rumen microbiota using the 16S rRNA sequencing approach, which is culture independent and enables a detailed characterization of the prokaryotic communities in the rumen (McCann *et al.*, 2014; Vasta *et al.*, 2019). In addition, the efficacy of polyphenols (tannins and flavonoids) in the above studies was tested using a single diet. The efficacies of polyphenols on rumen fermentation have been proved to be affected by diet type. For example, ruminal protein degradation was not affected by condensed tannin supplemented under concentrate‐based diet (Salami *et al.*, 2018) but inhibited under forage‐based diet (Tabacco *et al.*, 2006). Consequently, the effect of polyphenols on rumen fermentation and microbial composition should be evaluated in diets that reflect the types of feeds used in ruminant animals (Yáñez‐Ruiz *et al.*, 2016). Moreover, polyphenols such as rutin, naringin and quercitrin have been suggested to be readily degraded in the rumen (McSweeney *et al.*, 2001). Therefore, we hypothesized that resveratrol may be partly degraded after 24 h of *in vitro* fermentation in both high‐concentrate (HC) and high‐forage (HF) diets. Moreover, we hypothesized that resveratrol may differentially affect rumen fermentation between HC and HF diets, due to the interactive effect of resveratrol and diet on prokaryotic community composition.

In the current study, we investigated the effect of supplementation of resveratrol on *in vitro* methane production, rumen fermentation and prokaryotic communities using the 16S rRNA sequencing method under two different diets (forage‐based and concentrate‐based) and two time points (12 and 24 h) that reflect the types of diet of fattening lambs and feeding regimens in practice respectively. *In vitro* fermentation techniques involve the incubation of substrates (usually the diets) with a rumen fluid, which have been widely used to evaluate the effect of polyphenols such as tannins (Jayanegara *et al.*, 2015; Witzig *et al.*, 2018) and flavonoids (Oskoueian *et al.*, 2013; Seradj *et al.*, 2014) on VFA production and methane production. Although the results obtained from *in vitro* fermentation techniques cannot completely reflect or replace those obtained from *in vivo* studies (Benchaar *et al.*, 2008; Oh and Hristov, 2016), they offer a rapid and less expensive alternative to evaluate nutrient utilization *in vivo* (Rymer *et al.*, 2005). Given that animals are offered diets or supplements once or twice daily in practice, evaluating the degradation of polyphenols 12 or 24 h after feeding is essential to ensure that the active components are still available.

## Results

### Gas production and rumen fermentation parameters

**Fig. 1:**
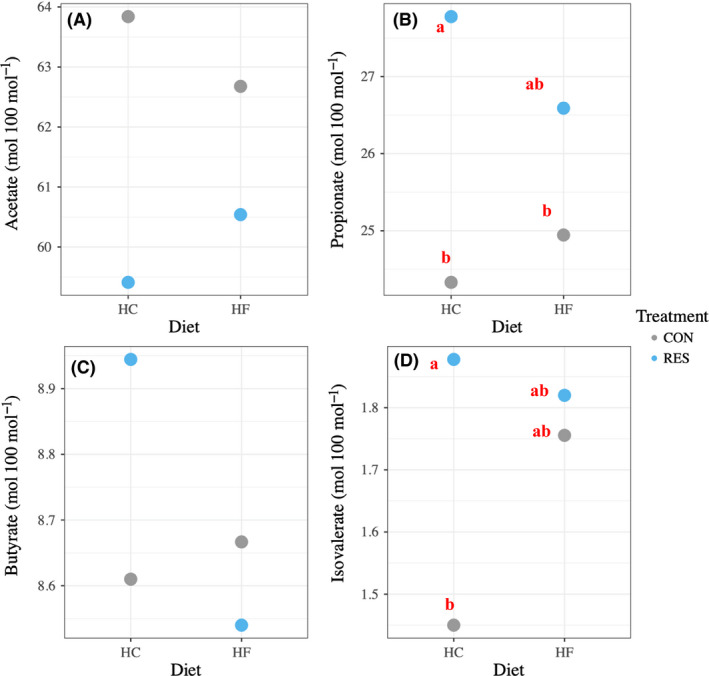
Interactive effect (P ≤ 0.05) of treatment (CON and RES) and diet (HC and HF) on the molar proportion of (A) acetate, (B) propionate, (C) butyrate and (D) isovalerate. CON and RES mean diet not supplemented or supplemented with resveratrol respectively. HC and HF mean high‐concentrate and high‐forage diets respectively. ‘a’ and ‘b’ in (B) and (D) means differ significantly (*P* ≤ 0.05).

Total gas production (ml per mg of dry matter degraded) was interactively affected by diet and time (*P* = 0.040) (Table S1). Treatment and time (*P* = 0.047) and diet and time (*P* < 0.001) interactively affected methane production (ml per mg of dry matter degraded). Total VFA concentration (mM) was affected by the interaction between treatment and time (*P* = 0.001). Treatment and diet interactively affected the molar proportion of acetate (*P* = 0.037) and isovalerate (*P* < 0.001) and tended to affect that of propionate (*P* = 0.081) and butyrate (*P* = 0.084) (Fig. 1) (Table S1). In brief, resveratrol inhibited methane production, decreased the molar proportion of acetate and increased that of propionate, regardless of diet type or fermentation time.

### Quantification of resveratrol and metabolites

Concentrations of resveratrol and its potential metabolites, including dihydroresveratrol, piceid and lunularin, were determined after 12 and 24 h of *in vitro* fermentation. The mass spectrum of resveratrol (retention time or Tr = 2.76 min) showed [M^−^H]^−^ion at m/z 227 (Fig. S1A) and dihydroresveratrol (Tr = 2.76 min) at m/z 229 (Fig. S1B). However, the mass spectrum of either piceid (Fig. S1C) or lunularin (Fig. S1D) was not detected. Thus, dihydroresveratrol was the only metabolite of resveratrol detected, and its concentration was higher after 24 h than after 12 h of fermentation (Table S2).

### Sequencing reads and amplicon sequence variants (ASVs)

Sequencing of the bacterial 16S rRNA gene of 40 samples resulted in 4 306 615 total reads, with an average of 107 665 ± 30 795 reads per sample. After quality control and the removal of potential contaminations, the remaining 3 003 315 (69.7%) reads were collapsed into 25 876 ASVs, with an average of 75 082 ± 3486 reads and 928 ± 25 ASVs per sample based on a 99% nucleotide sequence similarity (Table S3). Sequencing of the archaea 16S rRNA gene of the same 40 samples resulted in 1 915 922 total sequence reads. After quality control, the remaining 881 712 (46.0%) sequence reads were collapsed into 98 ASVs, with an average of 22,042 ± 1,588 sequence reads per sample based on a 99% nucleotide sequence similarity (Table S3). The ASVs were further used for the calculation of diversity (alpha and beta) and taxonomy analysis at the phylum and genus levels.

### Alpha and beta diversity metrics of bacteria

**Fig. 2:**
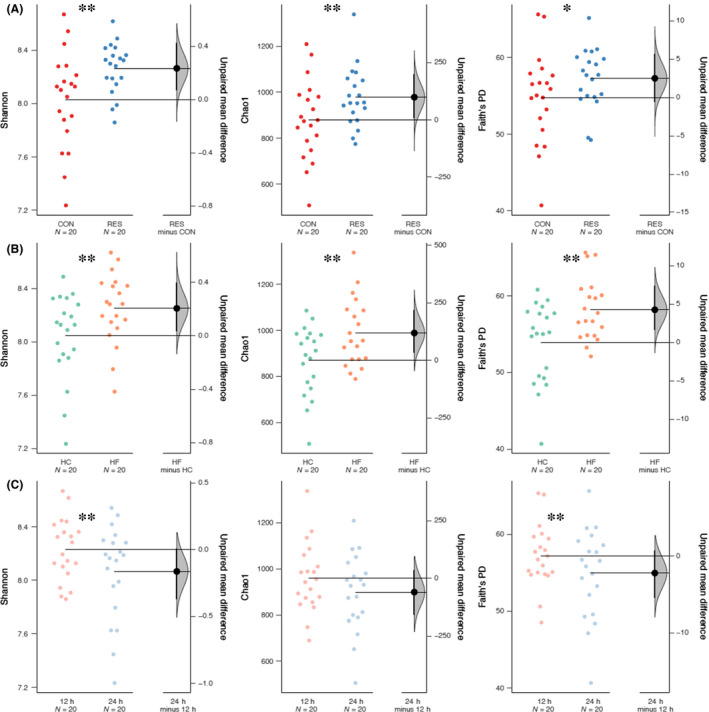
Comparison of the effect of (A) treatment (CON vs. RES), (B) diet (HC and HF) and (C) time (12 and 24 h) on bacterial alpha diversity including Shannon, Chao1 and Faith’s phylogenetic diversity (PD). Statistical analyses were done by Aligned Rank Transform procedure (‘**’ represents *P* ≤ 0.05, and ‘*’ represents 0.05 < *P* ≤ 0.10). The Gardner–Altman plots showed the effect size represented by mean difference between ‘CON’ and ‘RES’, ‘HC’ and ‘HF’ and ‘12’ and ‘24 h’ groups respectively (right panel). In the Gardner–Altman plots, the 95% confidence interval of the mean difference between ‘CON’ and ‘RES’, ‘HC’ and ‘HF’ and ‘12 h’ and ‘24 h’ groups is illustrated by the black vertical line. CON and RES mean diet not supplemented or supplemented with resveratrol respectively. HC and HF mean high‐concentrate and high‐forage diets respectively.

Treatment significantly affected the Shannon index (*P* = 0.011), Chao1 (*P* = 0.050) and Faith’s phylogenetic diversity (PD) index (*P* = 0.095) (Fig. 2A; Table S4). Similarly, diet significantly affected the Shannon index (*P* = 0.009), Chao1 (*P* = 0.022) and Faith’s PD index (*P* = 0.012) (Fig. 2B; Table S4). Fermentation time only affected the Shannon index (*P* = 0.050) (Fig. 2C; Table S4).

**Fig. 3:**
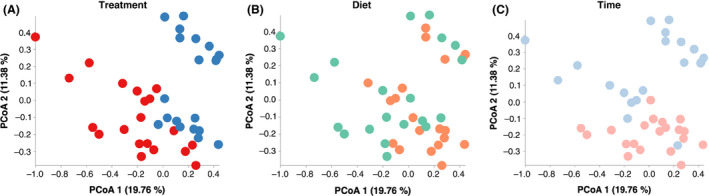
Principal coordinate analysis (PCoA) plots based on the unweighted UniFrac distances showed distinct clusters in the bacterial structure between (A) treatment (CON vs. RES), (B) diet (HC vs. HF) and (C) time (12 vs. 24 h). Samples belonging to different treatments (CON: blue, RES: red), diets (HC: green, HF: orange) and times (12 h: light blue; 24 h: light red) are differentiated by colours. CON and RES mean diet not supplemented or supplemented with resveratrol respectively. HC and HF mean high‐concentrate and high‐forage diets respectively.

**Fig. 4:**
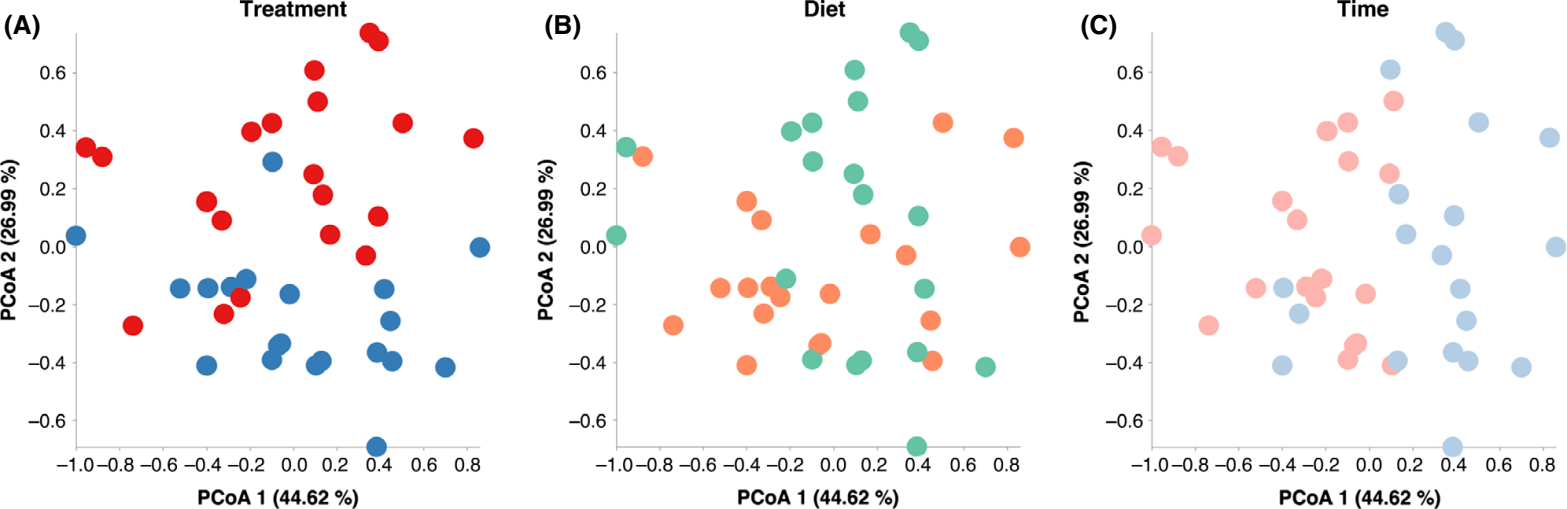
Principal coordinate analysis (PCoA) plots based on the weighted UniFrac distances showed distinct clusters in the bacterial structure between (A) treatment (CON vs. RES), (B) diet (HC vs. HF) and (C) time (12 vs. 24 h). Samples belonging to different treatments (CON: blue, RES: red), diets (HC: green, HF: orange) and times (12 h: light blue; 24 h: light red) are differentiated by colours. CON and RES mean diet not supplemented or supplemented with resveratrol respectively. HC and HF mean high‐concentrate and high‐forage diets respectively.

The principal coordinate analysis (PCoA) based on unweighted UniFrac distances did not show clear separation of bacterial profiles by treatment (*R* ^2^ = 0.122, *P* < 0.001; Fig. 3A), diet type (*R* ^2^ = 0.061, *P* < 0.001; Fig. 3B) or fermentation time (R^2^ = 0.090, *P* < 0.001; Fig. 3C) (Table S5). Similarly, the PCoA based on weighted UniFrac distances did not show clear separation of bacterial profiles by treatment (*R* ^2^ = 0.162, *P* < 0.001; Fig. 4A), diet (*R* ^2^ = 0.079, *P* = 0.003; Fig. 4B) or time (*R* ^2^ = 0.221, *P* < 0.001; Fig. 4C) (Table S5). In sum, bacterial diversity was more significantly affected by a single factor (e.g. treatment, diet or time) than the interaction of those factors.

### Alpha and beta diversity metrics of archaea

The Shannon index was interactively affected by treatment and time (*P* = 0.001) (Table S4). The PCoA based on weighted UniFrac distances did not show clear separation of archaea profiles by treatment (*R* ^2^ = 0.411, *P* < 0.001; Fig. S2A), diet (*R* ^2^ = 0.062, *P* = 0.009; Fig. S2B) or fermentation time (*R* ^2^ = 0.062, *P* = 0.013; Fig. S2C). Treatment and diet (*R* ^2^ = 0.042, *P* = 0.026) and treatment and time (*R* ^2^ = 0.092, *P* = 0.001) interactively affected the archaea profiles (Table S5).

### Effect of resveratrol on the bacterial community

A total of 13 bacterial phyla were detected (average relative abundance > 0.1%), with *Bacteroidetes* (46.8 ± 0.88%) being the predominant phylum, followed by *Firmicutes* (36.5 ± 0.69%), *Proteobacteria* (6.22 ± 0.40%) and *Synergistetes* (5.93 ± 0.27%). The relative abundance of *Proteobacteria* (7.71 ± 0.57% vs. 4.72 ± 0.32%, *P* < 0.001) was higher, while that of *Synergistetes* (5.43 ± 0.32% vs. 6.44 ± 0.40%, *P* = 0.010) was lower in the RES than in the CON group (Table S6). Treatment and diet interactively affected the relative abundance of *Chloroflexi* (*P* = 0.008), *Fibrobacteres* (*P* = 0.044), *Lentisphaerae* (*P* = 0.029) and *Patescibacteria* (*P* = 0.047). Treatment and time interactively affected the relative abundance of *Cyanobacteria* (*P* = 0.033) and *Spirochaetes* (*P* < 0.001). Treatment, diet and time interactively affected the relative abundance of *Tenericutes* (*P* = 0.006).

**Fig. 5:**
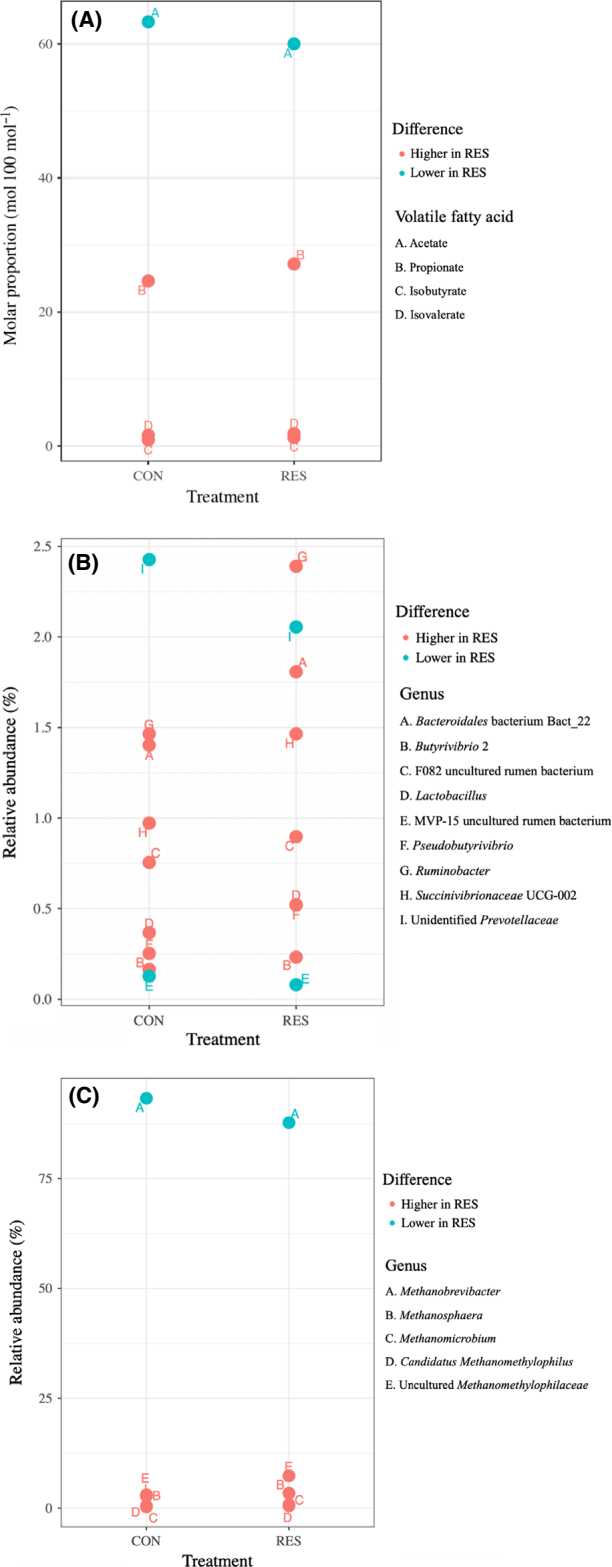
Difference in the (A) molar proportion of acetate, propionate, isobutyrate and isovalerate, (B) relative abundance of nine bacterial genera and (C) relative abundance of five archaea genera between CON and RES groups. CON and RES mean diet not supplemented or supplemented with resveratrol respectively. Red and green colours indicate the molar proportion/relative abundance is higher and lower in RES than in the CON group respectively (*P* ≤ 0.05).

A total of 85 bacterial genera were detected using the same cut‐off mentioned above (Table S7). *Prevotella* 1 was the predominant genus (24.1 ± 0.83%), followed by the genera belonging to *Quniella* (14.2 ± 0.50%) and *Fretibacterium* (5.54 ± 0.27%) in all samples. The relative abundance of *Bacteroidales* bacterium Bact_22 (1.81 ± 0.13% vs. 1.40 ± 0.13%, *P* = 0.044), F082 uncultured rumen bacterium (0.90 ± 0.06% vs. 0.60 ± 0.05%, *P* = 0.025), *Butyrivibrio* 2 (0.23 ± 0.02% vs. 0.17 ± 0.02%, *P* = 0.050), *Ruminobacter* (2.39 ± 0.34% vs. 1.47 ± 0.25%, *P* = 0.013) and *Succinivibrionaceae* UCG‐002 (1.47 ± 0.22% vs. 0.97 ± 0.18%, *P* = 0.013) was higher, whereas that of unidentified *Prevotellaceae* (2.06 ± 0.17% vs. 2.43 ± 0.16%, *P* = 0.049) and an uncultured bacterium belonging to phylum *Spirochaetes* (0.08 ± 0.01% vs. 0.13 ± 0.01%, *P* = 0.046) was lower in the RES than in the CON group (Fig. 5B).

**Fig. 6:**
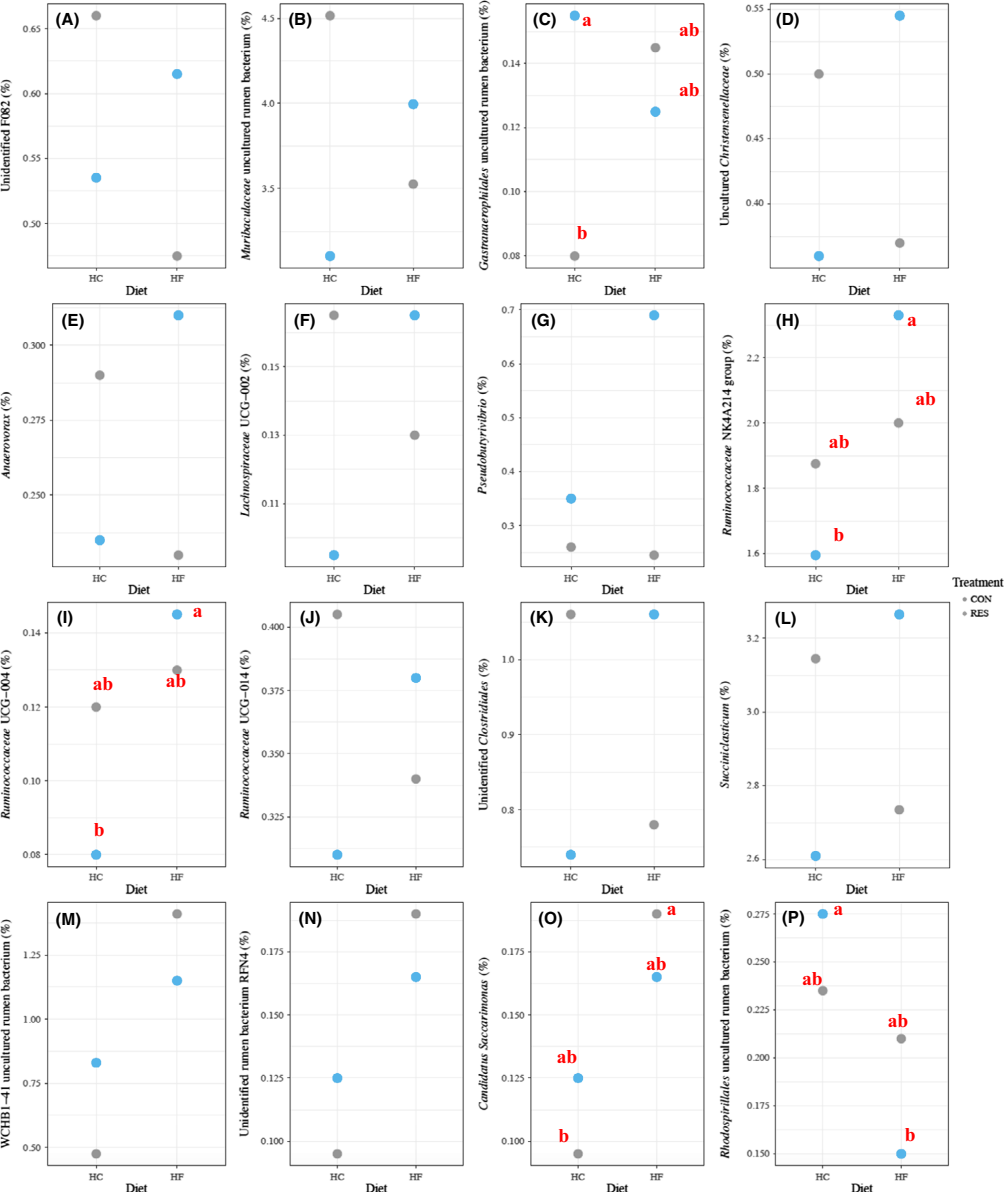
Interactive effect (*P* ≤ 0.05) of treatment (CON and RES) and diet (HC and HF) on the relative abundance of (A) Unidentified F082, (B) *Muribaculaceae* uncultured rumen bacterium, (C) *Gastranaerophilales* uncultured rumen bacterium, (D) Uncultured *Christensenellaceae*, (E) *Anaerovorax*, (F) *Lachnospiraceae* UCG‐002, (G) *Pseudobutyrivibrio*, (H) *Ruminococcaceae* NK4A214 group, (I) *Ruminococcaceae* UCG‐004, (J) *Ruminococcaceae* UCG‐014, (K) Unidentified *Clostridiales*, (L) *Succiniclasticum*, (M) WCHB1‐41 uncultured rumen bacterium, (N) Unidentified rumen bacterium RFN4, (O) *Candidatus Saccarimonas*, (P) *Rhodospirillales* uncultured rumen bacteria. CON and RES mean diet not supplemented or supplemented with resveratrol respectively. HC and HF mean high‐concentrate and high‐forage diets respectively. ‘a’ and ‘b’ in (C), (H), (I), (O) and (P) means differ significantly (*P* ≤ 0.05).

Treatment and diet interactively affected the relative abundance of unidentified F082 (*P* = 0.050), *Muribaculaceae* uncultured rumen bacterium (*P* = 0.050), *Gastranaerophilales* uncultured rumen bacterium (*P* = 0.009), *Lachnospiraceae* UCG‐002 (*P* = 0.006), *Pseudobutyrivibrio* (*P* = 0.006), *Ruminococcaceae* NK4A214 group (*P* = 0.019), *Ruminococcaceae* UCG‐004 (*P* = 0.026), *Ruminococcaceae* UCG‐014 (*P* = 0.029), unidentified *Clostridiales* (*P* = 0.018), *Succiniclasticum* (*P* = 0.006), uncultured WCHB1‐41 (*P* < 0.001), unidentified rumen bacterium RFN4 (*P* = 0.036), *Candidatus Saccharimonas* (*P* = 0.047) and *Rhodospirillales* uncultured rumen bacterium (*P* = 0.044) (Fig. 6; Table S7). Notably, the relative abundance of *Gastranaerophilales* uncultured rumen bacterium was higher (*P* = 0.029; Fig. 6C) in the RES than in the CON group only for the HC diet. The relative abundance of *Ruminococcaceae* NK4A214 group (*P* = 0.003; Fig. 6H), *Ruminococcaceae* UCG‐004 (*P* = 0.002; Fig. 6I) and *Candiadatus Saccharimonas* (*P* = 0.042; Fig. 6O) was higher, whereas that of *Rhodospirillales* uncultured rumen bacterium was lower (*P* = 0.007; Fig. 6P) in the HF than HC diet only for the RES group.

Treatment and time interactively affected the relative abundance of *Lactobacillus* (*P* = 0.040), *Pseudobutyrivibrio* (*P* = 0.004) and 0319‐6G20 uncultured rumen bacterium (*P* = 0.024) (Table S7).

**Fig. 7:**
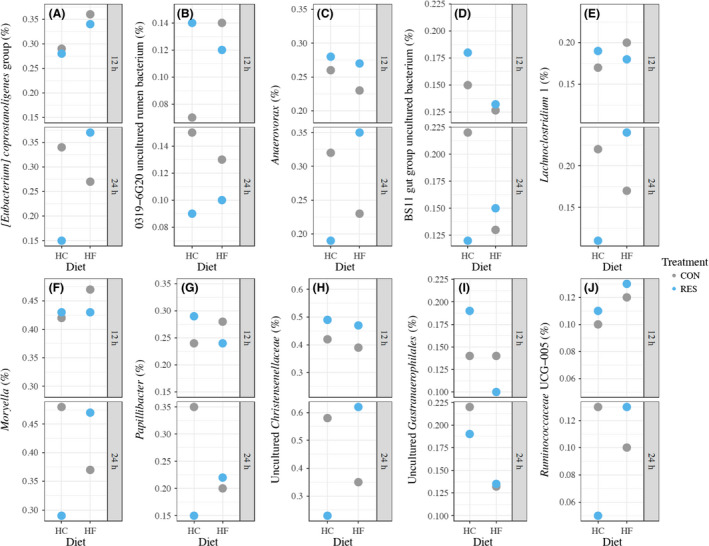
Interactive effect (*P* ≤ 0.05) of treatment (CON and RES), diet (HC and HF) and time (12 and 24 h) on the relative abundance of 10 bacterial genera (A) [*Eubacterium*] *coprostanoligenes* group, (B) 0319‐6G20 uncultured rumen bacterium, (C) *Anaerovorax*, (D) BS11 gut group uncultured bacterium, (E) *Lachnoclostridium* 1, (F) *Moryella*, (G) *Papillibacter*, (H) uncultured *Christensenellaceae*, (I) unidentified *Gastranaerophilales*, (J) *Ruminococcaceae* UCG‐005. CON and RES mean diet not supplemented or supplemented with resveratrol respectively. HC and HF mean high‐concentrate and high‐forage diets respectively.

Treatment, diet and time interactively affected the relative abundance of 10 bacterial genera, including [*Eubacterium*] *coprostanoligenes* group (*P* = 0.044), 0319‐6G20 uncultured rumen bacterium (*P* = 0.024), *Anaerovorax* (*P* = 0.040), BS11 gut group uncultured bacterium (*P* = 0.024), *Lachnoclostridium* 1 (*P* = 0.024), *Moryella* (*P* = 0.034), *Papillibacter* (*P* = 0.014), uncultured *Christensenellaceae* (*P* = 0.014), unidentified *Gastranaerophilales* (*P* = 0.002) and *Ruminococcaceae* UCG‐005 (*P* = 0.039) (Fig. 7; Table S7).

In summary, the taxonomic composition of bacteria was more significantly affected by treatment at the phylum level and by two‐way (treatment and diet) or three‐way (treatment, diet and time) interactions at the genus level.

### Effect of resveratrol on the archaea community

Five archaea genera, including *Methanobrevibacter* (90.5 ± 0.69%), *Methanosphaera* (3.08 ± 0.09%), *Methanomicrobium* (0.63 ± 0.09%), *Candidatus Methanomethylophilus* (0.42 ± 0.04%) and uncultured *Methanomethylophilaceae* (5.21 ± 0.58%), were detected. The relative abundance of *Methanosphaera* (3.42 ± 0.09% vs. 2.75 ± 0.13%, *P* < 0.001) and *Candidatus Methanomethylophilus* (0.53 ± 0.07% vs. 0.31 ± 0.04%, *P* = 0.003) was higher in the RES than in the CON group (Fig. 5C; Table S8). Treatment and diet interactively affected the relative abundance of *Methanosarcina* (*P* = 0.039). Treatment and time interactively affected the relative abundance of *Methanobrevibacter* (*P* = 0.001) and uncultured *Methanomethylophilaceae* (*P* = 0.002). Treatment, diet and time interactively affected the relative abundance of *Methanomicrobium* (*P* < 0.001) (Table S8). In sum, the taxonomic composition of archaea was more significantly affected by treatment alone at the genus level.

## Discussion

*In vitro* fermentation techniques have been extensively used to evaluate the effect of diets or additives on rumen fermentation and methane production (Durmic *et al.*, 2010). These techniques allow for the controlling of experimental conditions more precisely than do *in vivo* experiments (Makkar, 2004) and can thus be used for screening and informing on the suitability of further *in vivo* studies (Yáñez‐Ruiz *et al.*, 2016). With *in vitro* fermentation techniques, how supplements (e.g. resveratrol) affect fermentation, methane production and microbiota under different circumstances can be evaluated simultaneously.

In the current study, lower total VFA concentration in response to resveratrol was observed, regardless of diet type or fermentation time. The inhibitory effect of polyphenols on rumen fermentation has also been reported. For example, Becker and Wikselaar (2011) observed that supplementation of resveratrol inhibited total VFA concentration using Hydrogen Release Compound eXtended® (HRC‐X) as a substrate. The total VFA concentration was also significantly decreased in the presence of flavonoids, including flavone, myricetin and kaempferol (Oskoueian *et al.*, 2013). However, none of these studies considered the effect of diet type or fermentation time and thus limited our understanding of how those factors interact with rumen microbiota.

The higher molar proportion of propionate may be explained by the higher relative abundance of two taxa belonging to family *Succinivibrionaceae* (*Succinivibrionaceae* UCG‐002 and *Ruminobacter*) and two taxa belonging to the order *Bacteroidales* (bacterium Bact_22 and an uncultured F082 rumen bacterium) in the RES than in the CON group. *Succinivibrionaceae* ferments carbohydrates to produce succinate, a precursor of propionate (Pope *et al.*, 2011). Several bacterial taxa belonging to the order *Bacteroidales* were reported to be positively correlated with propionate production in the rumen of cattle (Wallace *et al.*, 2019). In addition, higher relative abundance of *Lactobacillus* was detected under RES, which could also contribute to the higher molar proportion of propionate. A study suggested that an increased *in vitro* propionate production was associated with the growth of *Lactobacillus mucosae* (Mamuad *et al.*, 2017). Molar proportion of butyrate was not different between the two treatments. One possible reason is that butyrate accounts for a relatively smaller part of the total VFA, and thus its change may be masked by the changes of acetate or propionate.

Treatment and diet interactively affected the relative abundance of an uncultured rumen bacterium (*Gastranaerophilales* family). *Gastranaerophilales* belongs to class *Melainabacteria*, which is capable of fermenting a range of sugars (e.g. glucose, starch and hemicellulose) into butyrate in the gut of herbivores (Di Rienzi *et al.*, 2013). Therefore, we speculate that the supplementation of resveratrol may facilitate the growth of *Gastranaerophilales* when the diets are rich in readily fermentable carbohydrates (e.g. HC diet). Higher relative abundance of *Candidatus Saccharimonas*, a potential cellulose utilizer (Opdahl *et al.*, 2018), was observed in the HF diet in response to resveratrol, suggesting that resveratrol could promote its growth when the diets are rich in fibre. In contrast, the relative abundance of an uncultured rumen bacterium (*Rhodospirillales* family) was lower in response to resveratrol in the HF diet. A single‐contig annotated as uncultured *Rhodospirillales* was recently reported to encode predicted CAZymes, including glycosyl transferases and glycosyl hydrolases (Stewart *et al.*, 2019). Taken together, resveratrol may affect the growth of certain bacterial taxa in a diet‐specific manner and the underlying mechanisms need further investigation.

The 10 bacterial genera whose relative abundance was affected by three‐way interaction (treatment, diet and time) contributed to less than 3% of the total bacterial community. Among them, *Lachnoclostridium* (Ravachol *et al.*, 2015), *Moryella* (Pitta *et al.*, 2014) and *Papillibacter* (Zhang *et al.*, 2014) were reported to be associated with the degradation of polysaccharide in the rumen. Interestingly, the relative abundance of the three genera was affected by the three‐way interaction in a similar pattern. These results suggest that the effect of resveratrol on rumen fermentation and microbial profiles can be affected by multiple factors. Therefore, the conclusions drawn in previous studies of plant extracts in which only treatment effect was tested should be interpreted with caution.

The genera *Methanobrevibacter* and *Methanosphaera* represented about 98% of the archaeal community in lactating cows (Kumar *et al.*, 2015). *Methanosarcinales* and *Methanomicrobiales* clades were found to make up less than 1% of the total archaea community in sheep rumen (Sneilling *et al.*, 2014). Our findings were consistent with those previous results. The mitigation of methane production by tannins in *vitro* has been demonstrated to be associated with the decrease in the abundance of *Methanobrevibacter* (Saminathan *et al.*, 2016; Witzig *et al.*, 2018). Therefore, the lower methane production may also be associated with lower relative abundance of *Methanobrevibacter* in the RES group. Tannins were found to completely inhibit methane production by limiting the growth of *Methanobrevibacter* strains YLM‐1 and DSM1093 (Tavendale *et al.*, 2005). Future studies are needed to investigate which exact species/strain(s) of *Methanobrevibacter* is/are sensitive to the supplementation of resveratrol.

The microbial metabolites of resveratrol include dihydroresveratrol, piceid and lunularin in human, rat and *in vitro* studies (Jung *et al.*, 2009; Bode *et al.*, 2013; Etxeberria *et al.*, 2015). However, we were only able to detect dihydroresveratrol in the *in vitro* fermentation system. Several microbes have been reported to metabolize resveratrol. For example, *Eggerthella lenta* ATCC 43055 (Jung *et al.*, 2009), *Slackia equolifaciens* and *Adlercreutzia equolifaciens* (Bode *et al.*, 2013), which belong to *Coriobacteriaceae*, are responsible for the bioconversion of resveratrol to dihydroresveratrol. In the current study, three genera from family *Coriobacteriaceae*, namely *Atopobium*, *Olsenella* and an unclassified genus belonging to family *Eggerthellaceae*, were detected. Whether those genera play a role in the bioconversion of resveratrol remains unclear. Nevertheless, our result showed that resveratrol was not completely metabolized after 24 h of *in vitro* fermentation. This outcome may support the use of resveratrol as a feed additive on a daily basis in ruminants. In addition to microbe‐induced bioconversion, resveratrol has been reported to be stable in acid media (pH under 6.8), in which temperature had a minimal effect on its degradation (Zupančič *et al.*, 2015). Although most of the diets had a pH lower than 6.8 in the current study, the normal ruminal pH in sheep is 5.5–7.0 depending on the diet (Jasmin *et al.*, 2011). Therefore, *in vivo* studies are needed to further investigate the kinetics of resveratrol in the rumen and the exact microbial species/strain that interacts and metabolizes resveratrol. Note that the dose of resveratrol needed for *in vivo* studies may be different from that used in the current study. According to a meta‐analysis, the dose of phytonutrients used *in vitro* was 0.03–500 g kg^−1^ of the dietary dry matter (DM) (Klevenhusen *et al.*, 2012), whereas the doses used for *in vivo* studies were 0.02–0.75 (small ruminants), 0.04–0.25 (beef cattle) and 0.01–0.43 (dairy cows) g per kg of dietary DM depending on the animals (Khiaosa‐ard and Zebeli, 2013). In this regard, animal studies are needed to validate the proximal dose of resveratrol needed for the effective manipulation of rumen fermentation and methane production.

In conclusion, the current study suggests that resveratrol can inhibit ruminal methane production regardless of diet type, highlighting the feasibility of using it for mitigating methane emission from sheep fed various types of diets. The molar proportion of propionate may be associated with the higher relative abundance of taxa that facilitate propionate production (e.g. *Gastranaerophilales*) in response to resveratrol only in the HC diet. The relative abundance of 10 bacterial genera was affected by three‐way interactions of diet, fermentation time and supplements, suggesting that multiple factors should be considered in studies of the effect of polyphenol on rumen microbiota. Resveratrol was partly converted to dihydroresveratrol at 12 and 24 h of fermentation, and the specific taxa responsible for the degradation of resveratrol should be identified. Animal experiments with multiple factors (e.g. diet and time) taken into consideration are needed to determine the interaction between resveratrol or other polyphenols and rumen microbiota.

## Experimental procedures

### In vitro fermentation system

Six rumen‐cannulated sheep were fed with Chinese wildrye hay (DM: 93.5%, NDF: 58.9%, ADF: 28.7%) and a commercial concentrate (DM: 90.9%, NDF: 30.6%, ADF: 11.6%; TMR no. 1, Wellhope Agri‐Tech Joint Stock Co., Ltd., Liaoning, China). The main ingredients of the commercial concentrate were corn, soybean meal, extruded soybean, cottonseed meal, DDGS and mineral/vitamin premix. Three sheep were fed hay and concentrate at a ratio of 3:7 (HC), and the other three sheep were fed hay and concentrate at a ratio of 7:3 (HF). The chemical composition of the two diets is shown in Table 1. All sheep were fed once daily at 8:00 in the morning, and rumen fluid was taken immediately before the morning feeding. The management and care of the rumen‐cannulated sheep were performed according to the protocols approved by the Feed Research Institute of Chinese Academy of Agricultural Sciences.

**Table 1:** Chemical composition of the diet (air‐dry basis).

Item	Diet	
	HC	HF
DM (%)	87.8	88.5
CP (% of DM)	17.1	13.6
GE (% of DM)	16.8	16.3
EE (% of DM)	2.46	2.43
NDF (% of DM)	25.1	46.6
ADF (% of DM)	12.7	25.4

ADF, acid detergent fibre; CP, crude protein; DM, dry matter; EE, ether extract; GE, gross energy; NDF, neutral detergent fibre.

HC and HF mean high‐concentrate and high‐forage diets respectively.

**Fig. 8:**
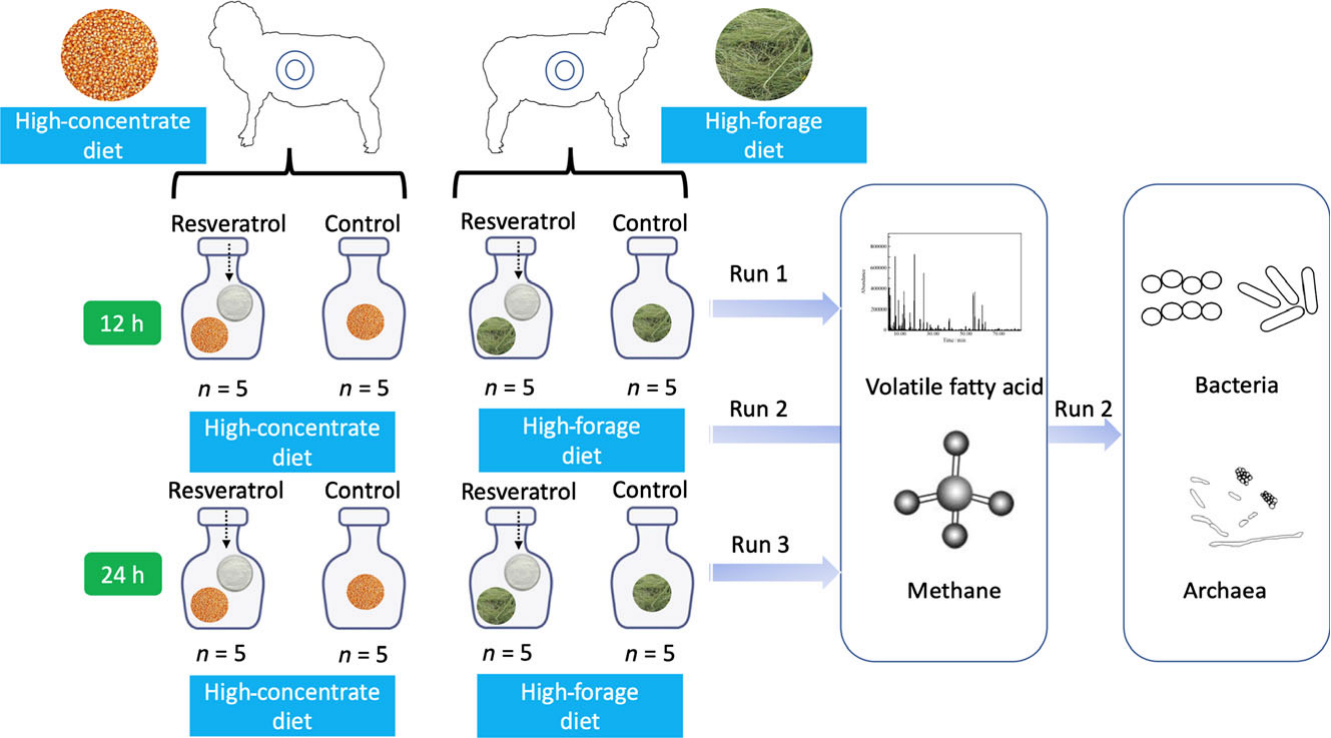
Experimental design of the current study.

Rumen fluid was filtered over four layers of cheesecloth and collected in a pre‐warmed (39°C) thermostated flask (4 l) filled with CO_2_. Rumen fluid and artificial saliva (McDougall, 1948) were combined at a ratio of 1:2 (v/v), and a 30 ml aliquot of the buffered solution was piped to a glass syringe (volume: 100 ml, diameter: 32 mm; Häberle LABORTECHNIK, Lonsee‐Ettlenschieß, Germany), in which a mixture of 300 mg of diet with 25 mg of resveratrol (RES) (purity: 98%; World‐Way Biotech Inc., Hunan, China) or without resveratrol (CON) had already been placed. The diets used in the current study were exactly the same as the diet (HF and HC) for sheep. After shaking the syringe and removing the bubbles, the clip on the silicon tube (length: 50 mm, internal diameter: 5 mm) was closed, and the syringe was placed in a water bath maintained at 39ºC. The experiment was performed in three runs with five replicates per run. Gas, methane production, total VFA concentration and the molar proportion of acetate, propionate, butyrate, isobutyrate, valerate and isovalerate of CON and RES in both diets (HC and HF) were measured after 12 and 24 h of fermentation. In the second run of fermentation, 10 ml of ruminal fluid from each replicate was collected after 12 and 24 h of fermentation, respectively, and then stored at –80°C for further measuring of the microbial composition (Fig. 8).

### Measurement of methane production

The methane concentration of gas collected at 12 and 24 h of fermentation was measured by gas chromatography (Shimadzu GC‐2010; Shimadzu Corporation, Kyoto, Japan). Air samples (5 ml) were injected into a split injector port at 200°C with a split/splitless ratio of 1:10 and carrier gas (N_2_) at 29 kPa. A megabore HP‐MOLSIV column (film thickness: 30 m × 0.53 mm × 25 µm) was ran isothermally at 50°C. Methane was detected with a thermal conductivity detector, current at 48 mA, a negative mode and a N_2_ make‐up flow of 2.5 ml. An external standard with a known composition (H_2_ 5.2%, methane 9.7% and CO_2_ 15.0%) in N_2_ (BOC gases, Auckland, New Zealand) was injected every 2 h with each batch of headspace gas samples to quantify the methane composition. The peak areas were determined by automatic integration.

### Measurement of VFA concentration

The VFA concentration and molar proportion of each VFA at 12 and 24 h of fermentation were measured according to Zhang *et al. *(2016). Briefly, 1 ml of the rumen fluid filtrate was mixed with 25% metaphosphoric acid solution, which contained 2% 2‐ethyl butyrate, and then frozen at –20°C overnight. After thawing, the samples were centrifuged, and the supernatants were analysed by gas chromatography (SP‐3420, Beijing Analytical Instrument Factory, Beijing, China) using a column packed with 10% PEG‐20M and 2% H_3_PO_4_ (6 mm × 2 mm ID glass) with the following parameters: column temperature: 200°C, carrier gas: nitrogen, gas flow: 30 ml min^−1^, flame ionization detector temperature: 200°C, injector temperature: 200°C and injection volume: 0.6 µl.

### Quantification of resveratrol and metabolites

The standards of resveratrol (purity ≥ 98%; ANPEL Laboratory Technologies Inc., Shanghai, China), dihydroresveratrol (purity ≥ 98%; Yuanye Bio‐Technology Co., Ltd, Shanghai, China), piceid (purity ≥ 95%; Aladdin Co., Ltd., Shanghai, China), lunularin (purity ≥ 98%; Yake Chemistry Reagent Co., Ltd., Suzhou, Jiangsu, China), HPLC gradient grade acetonitrile, methanol, water and ammonia (Sigma‐Aldrich Corporation, Beijing, China) were used. The quantification of resveratrol along with its metabolites was performed using the Waters Acquity H‐Class UPLC system (Milford, MA, USA) equipped with an electrospray ion source and operated by the MassLynx4.1 software (Waters, Milford, MA, USA). The separation of resveratrol and dihydroresveratrol was conducted using a C18 reverse phase column (100 mm × 3 mm; particle size: 5 μm; Shimadzu Corporation), and the separation of piceid and 3,5,4’ trihydroxy‐trans‐stilbene was performed using a UPLC HSS T3 column (75 mm × 2.1 mm, particle size: 1.8 μm; Waters). The mobile phase consisted of acetonitrile and 0.05% (v/v) ammonia for the quantification of resveratrol and dihydroresveratrol, and the mobile phase consisted of methanol and 0.05% (v/v) ammonia for the quantification of piceid and 3,5,4’ trihydroxy‐trans‐stilbene. All solutions were degassed by sonication for 15 min at room temperature prior to use, and an online degassing was used during the analysis. The flow rate of the mobile phase was 0.4 ml min^−1^. All samples were filtered, and 2 μl was directly injected. The calibration curves were obtained by the linear regressing of the peak area of resveratrol and its metabolites against the known standard concentrations.

### Microbial DNA extraction and amplicon sequence

The extraction of the total DNA from each rumen fluid sample was conducted using the modified repeated bead‐beating method of Yu and Morrison (2004). DNA quantity and quality were further evaluated using a NanoDrop 1000 spectrophotometer (NanoDrop Technologies, Wilmington, DE, USA). To assess the microbial profiles, the bacterial V3‐V4 hypervariable region and the archaeal V6‐V8 hypervariable region of 16S rRNA genes were amplified respectively. For bacteria, the primers used were 338F (5′‐ ACTCCTACGGGAGGCAGCAG‐3′) and 806R (5′‐GGACTACHVGGGTWTCTAAT‐3′) (Dennis *et al.*, 2013), and for archaea, the primers used were Ar915aF (5′‐AGGAATTGGCGGGGGAGCAC‐3′) and Ar1386R (5′‐GCGGTGTGTGCAAGGAGC‐3′) (Henderson *et al.*, 2015). The polymerase chain reaction products were purified with the QIAEX II gel extraction kit (Qiagen Science, MD, USA), and the quality and quantity of purified amplicon were evaluated using the NanoDrop 1000 (NanoDrop Technologies, Wilmington, DE, USA) and the Picofluor Handheld Fluorometer using picogreen‐based chemistry (Quant‐iTTM PicoGreenTM dsDNA Reagent). The amplicon sequencing with a paired end was performed in Beijing Allwegene Tech Ltd. (Beijing, China) using the MiSeq platform (Illumina, 2 × 300 bp). The identified sequences from this study were deposited in the NCBI Sequence Read Archive (accession numbers: SRR9974839 to SRR9974918).

### Sequencing data analysis

The sequence data were analysed using the Quantitative Insight into Microbial Ecology 2 (QIIME2) platform (version 2019.7; Bolyen *et al.*, 2019). Briefly, the paired sequences were demultiplexed with a ‘demux’ plugin before being subjected to quality control using the ‘dada2’ plugin (Callahan *et al.*, 2016). The dada2‐based denoising identifies the amplicon sequence variants (ASVs), which infers the biological sequences prior to the introduction of amplification and sequencing errors in the samples (Callahan *et al.*, 2017). Taxonomy was assigned to the ASVs using a pre‐trained QIIME2‐compatible SILVA database (released in July 2019 and available at https://docs.qiime2.org/2019.7/data-resources/) with 99% identity for bacteria and archaea, and the taxonomies were assigned to the representative sequences. The potential contaminant sequences were removed using the ‘decontam’ R package (Davis *et al.*, 2018) according to the developer’s guide.

The alpha diversity indices, including Shannon, Chao1 and Faith’s PD, were calculated using the qiime2 ‘diversity’ plugin. The PcoA of the bacterial and archaea profiles based on either unweighted UniFrac or weighted UniFrac distance was conducted using the qiime2 ‘diversity’ plugin. Permutational analysis of variance (PERMANOVA) (Anderson, 2001) was performed to analyse the effect of treatment (CON and RES), diet (HC and HF), time (12 and 24 h) and their interactions on the bacterial and archaea profiles using the qiime2 ‘diversity’ plugin.

### Statistical analysis

Total gas production, methane production, total VFA concentration and molar proportion of acetate, propionate, butyrate, isobutyrate, valerate and isovalerate of the samples were analysed using a mixed model with repeated measures using the ‘lme4’ package in R (version 3.6.1). The difference in the alpha diversity indices, relative abundance of bacteria at the phylum and genus levels and relative abundance of archaea at the genus level were analysed using the aligned rank transform (ART) method, a non‐parametric approach that enables the analysis of multiple independent variables, interactions and repeated measures (Wobbrock *et al.*, 2011), using the ‘ARTool’ package in R (version 3.6.1). Samples in each run were considered as random effect, and treatment (CON and RES), diet (HC and HF) as well as time (12 and 24 h) were considered as fixed effect. A significant difference was declared at a *P* value ≤ 0.05 and tendencies at 0.05 < *P* ≤ 0.10.

## Conflict of interest

The authors declare no conflict of interest.

## Supporting information


**Fig. S1.** Quantification of (A) resveratrol, (B) dihydroresveratrol, (C) piceid and (D) lunularin in high‐concentrate and high‐forage diets at 12 and 24 h of fermentation.

**Fig.**

**Fig. S2.** Principal coordinate analysis plots based on the weighted UniFrac distances show distinct clusters in the archaea structure between (A) treatment (CON vs. RES), (B) diet (HC vs. HF) and (C) time (12 vs. 24 h). The samples belonging to different treatments (CON: blue, RES: red), diets (HC: green, HF: orange) and times (12 h: light blue; 24 h: light red) are differentiated by colour. CON and RES indicate the diet not supplemented and the diet supplemented with resveratrol, respectively. HC and HF indicate high‐concentrate diet and high‐forage diet, respectively.

**Table S1.** *In‐vitro* methane production, total volatile fatty acid (VFA) concentration and molar proportion of the individual VFAs after 12 and 24 h of fermentation.
**Table S2.** Quantification of resveratrol and its metabolites in high‐concentrate and high‐forage substrates after 12 and 24 h of fermentation.
**Table S3.** Sequencing reads and amplicon sequence variants of the prokaryotic communities.
**Table S4.** Alpha diversity of the prokaryotic communities after 12 and 24 h of fermentation.
**Table S5.** Permutational analysis of variance of the prokaryotic profiles using unweighted and weighted UniFrac distances.
**Table S6.** Taxonomic composition of the bacterial community at the phylum level after 12 and 24 h of fermentation.
**Table S7.** Taxonomic composition of the bacterial community at the genus level after 12 and 24 h of fermentation.
**Table S8.** Taxonomic composition of the archaea community at the genus level after 12 and 24 h of fermentation.
