# Supplementary figures and images for: Resveratrol affects in vitro rumen fermentation, methane production and prokaryotic community composition in a time‐ and diet‐specific manner
Source: Microb Biotechnol. 2020 Apr 15;13(4):1118–31. doi: 10.1111/1751-7915.13566 (PMC7264885; doi:10.1111/1751-7915.13566)

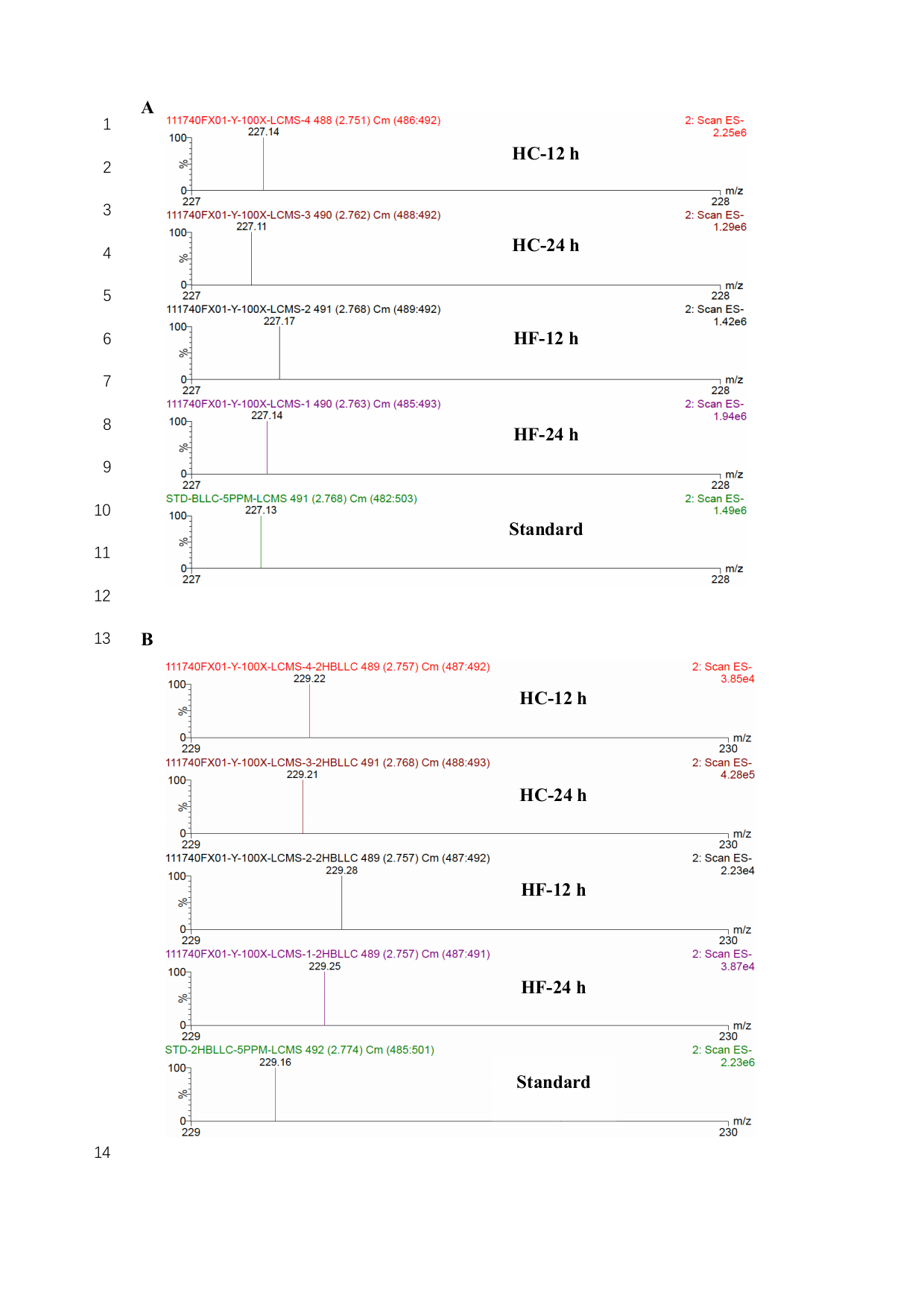

Supplement: Supplementary file 1 — Fig. S1. Quantification of (A) resveratrol, (B) dihydroresveratrol, (C) piceid and (D) lunularin in high‐concentrate and high‐forage diets at 12 and 24 h of fermentation. [file MBT2-13-1118-s001.tiff]

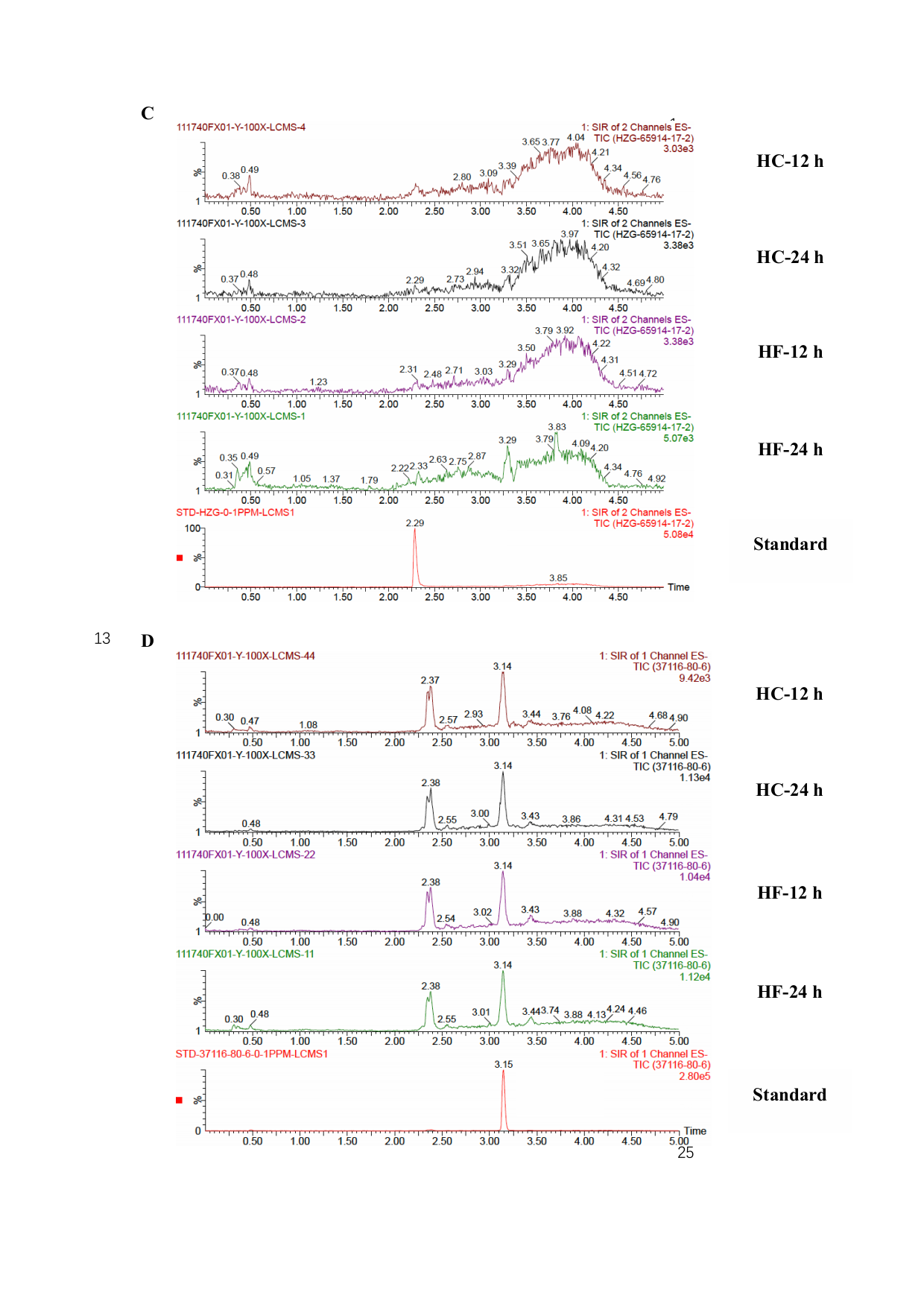

Supplement: Supplementary file 2 — Fig. [file MBT2-13-1118-s002.tiff]

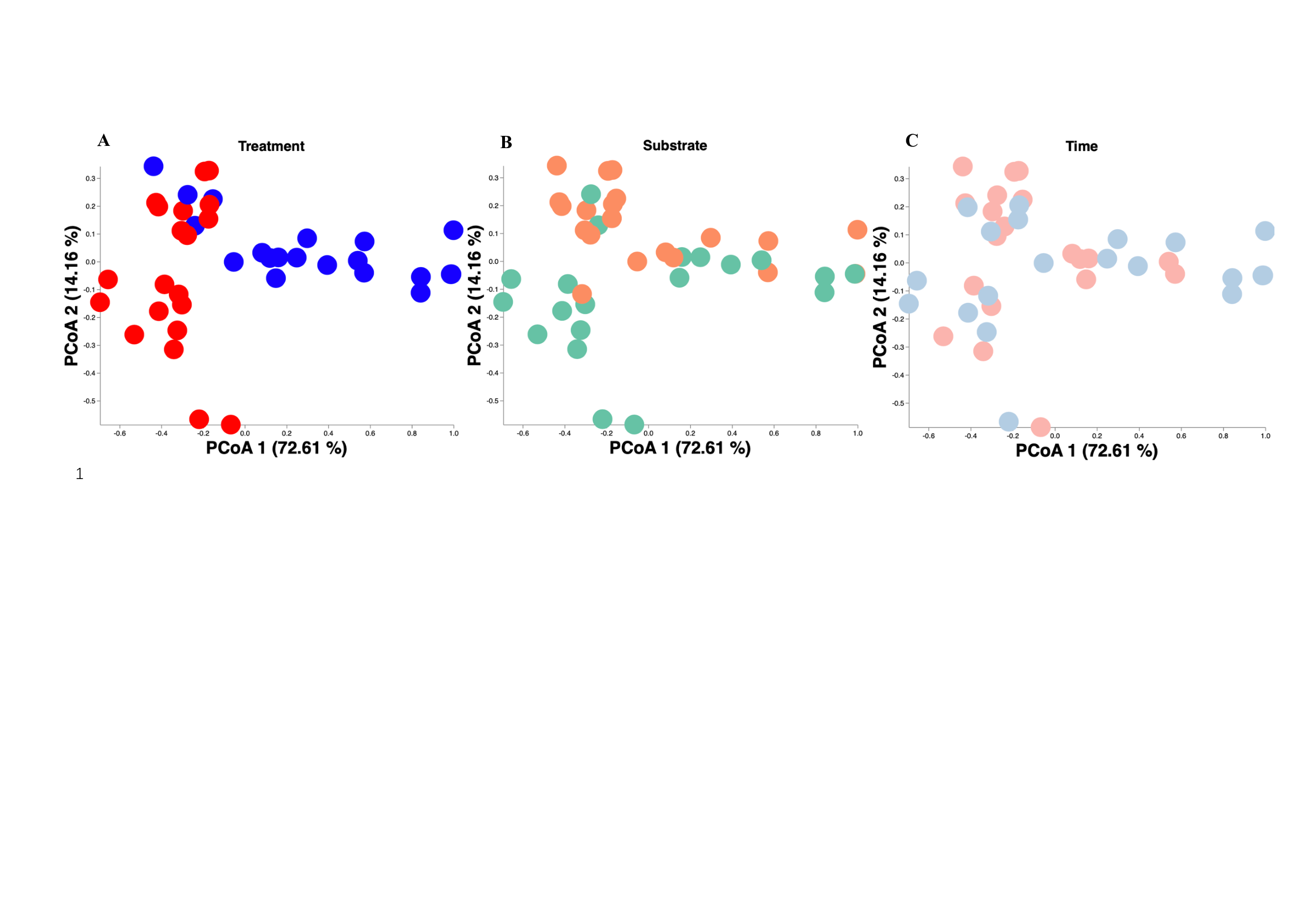

Supplement: Supplementary file 3 — Fig. S2. Principal coordinate analysis plots based on the weighted UniFrac distances show distinct clusters in the archaea structure between (A) treatment (CON vs. RES), (B) diet (HC vs. HF) and (C) time (12 vs. 24 h). The samples belonging to different treatments (CON: blue, RES: red), diets (HC: green, HF: orange) and times (12 h: light blue; 24 h: light red) are differentiated by colour. CON and RES indicate the diet not supplemented and the diet supplemented with resveratrol, respectively. HC and HF indicate high‐concentrate diet and high‐forage diet, respectively. [file MBT2-13-1118-s003.tiff]
